# Pathological and Clinical Features and Management of Central Nervous System Hemangioblastomas in von Hippel-Lindau Disease

**DOI:** 10.15586/jkcvhl.2014.12

**Published:** 2014-08-05

**Authors:** Hiroshi Kanno, Natsuki Kobayashi, Satoshi Nakanowatari

**Affiliations:** 1 Department of Neurosurgery, Yokohama City University School of Medicine; 2 Department of Neurosurgery, Yokosuka City Hospital, Yokosuka, Japan

## Abstract

Central nervous system (CNS) hemangioblastoma is the most common manifestation of von Hippel-Lindau (VHL) disease. It is found in 70–80% of VHL patients. Hemangioblastoma is a rare form of benign vascular tumor of the CNS, accounting for 2.0% of CNS tumors. It can occur sporadically or as a familial syndrome. CNS hemangioblastomas are typically located in the posterior fossa and the spinal cord. VHL patients usually develop a CNS hemangioblastoma at an early age. Therefore, they require a special routine for diagnosis, treatment and follow-up. The surgical management of symptomatic tumors depend on many factors such as symptom, location, multiplicity, and progression of the tumor. The management of asymptomatic tumors in VHL patients are controversial since CNS hemangioblastomas grow with intermittent quiescent and rapid-growth phases. Preoperative embolization of large solid hemangioblastomas prevents perioperative hemorrhage but is not necessary in every case. Radiotherapy should be reserved for inoperable tumors. Because of complexities of VHL, a better understanding of the pathological and clinical features of hemangioblastoma in VHL is essential for its proper management.

## Introduction

Von Hippel-Lindau (VHL) disease is inherited in an autosomal dominant pattern and characterized by the development of hemangioblastomas (HBs) of the central nervous system (CNS) and retina, renal cell carcinoma, pheochromocytoma, pancreatic and endolymphatic sac tumors. CNS HB is the most common VHL-associated lesion, and it is found in 70–80% of VHL patients. HB is a WHO grade 1 tumor, composed of stromal cells and abundant capillaries. Its cytogenesis remains uncertain, but recently it was suggested that HB originates from embryonic hemangioblast. The neurologic morbidity and mortality depend on HB’s location and multiplicity. Because of complexities of VHL, a deep understanding of clinical and pathological features of HB in VHL is essential (1).

## Histopathology and molecular markers

HBs are characterized histologically by two main components, large vacuolated stromal cells, and a rich capillary network composed of vascular endothelia and pericytes. The stromal cells represent the neoplastic component of the tumor, but the histogenesis remains uncertain. It has been suggested that the stromal cells are derived from hemangioblast progenitor cells (2, 3) and that the vascular cells represent reactive angiogenesis (4). The nuclei of the stromal cells vary in size with occasional atypical and hyperchromatic nuclei. Their most striking morphological feature is numerous lipid-containing vacuoles, characterizing the typical clear cell morphology. HB histologically mimics the clear cell type of renal cell carcinoma, but differential diagnosis can be made. Renal cell carcinoma commonly stains for markers including cytokeratin, EMA and pan-epithelial antigen, whereas HB does not stain for these markers.

In HB, the stromal cells and capillary endothelial cells significantly differ in their antigen expressing patterns. Stromal cells are commonly positive for neuron-specific enolase, neural cell-adhesion molecule (CD56), ezrin and vimentin (3). The capillary endothelial cells are commonly positive for CD 34 and CD 31(PECAM) (4). The stromal cells express high levels of epidermal growth factor receptor (EGFR), but the EGFR gene is not amplified (5). A subpopulation of the stromal cells also express transforming growth factor alpha (TGF-α), an EGFR ligand, which may suggest an autocrine TGF-α-EGFR loop (6). Vascular endothelial growth factor (VEGF) is highly expressed in stromal cells corresponding to endothelial expression of its receptors VEGFR-1 and -2 (7) and endothelial receptor Tie-1 (8). In addition to VEGF, erythropoietin and hypoxia inducible factor 2 alpha (HIF2-α) are highly expressed in the stromal cells (9, 10).

## Molecular mechanisms

The VHL gene was isolated by positional cloning at chromosome 3p25-26 and encodes a protein of 213 amino acids corresponding to a coding sequence of 639 nucleotides (11). The predicted protein contained an acidic pentameric repeat. The VHL gene has the characteristics of a classic tumor suppressor gene; i.e., loss of the wild type allele in CNS HB patients with VHL, and somatic mutations in sporadic CNS HB with a loss of heterozygosity (12–14). The VHL gene is expressed in a variety of tissues, in particular epithelial cells of the skin, the gastrointestinal, respiratory and urogenital tracts, and the endocrine and exocrine organs (14, 15 16). In the CNS, immunoreactivity for VHL protein is prominent in neurons, including Purkinje cells of the cerebellum (17, 18). Inactivation of the VHL gene in affected VHL family members is responsible for their genetic susceptibility to hemangioblastoma. The mechanism by which VHL protein causes neoplastic transformation has remained unclear.

**Figure 1.**
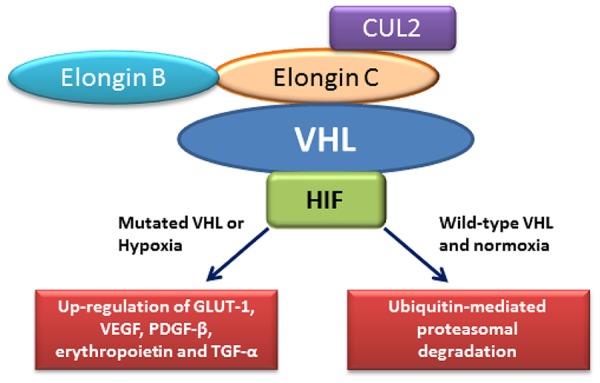
The interaction of VHL protein with HIF and other proteins including elongin B, elongin C and CUL2. A mutated VHL stabilizes HIF and leads to the up-regulation of many pro-angiogenic factors including GLUT-I, VEGF, PDGF-β, erythropoietin and TGF-α. A wild type VHL degrades HIF through ubiquitin-mediated pathway.

The VHL protein binds to elongins B and C, which activates transcription elongation by RNA polymerase H, and inhibits elongin (S HI) transcriptional activity (19), suggesting that the VHL protein may play an important role in the transcriptional regulatory network that controls tumorigenesis. The wild-type VHL protein regulates the expression of many hypoxia-induced genes such as vascular endothelial growth factor (Figure 1). The VHL protein inhibits the cellular expression of vascular endothelial growth factor, platelet-derived growth factor, and glucose transporter GLUT1 in hypoxic condition, but not in normoxic condition (19, 20). The VHL protein regulates the mRNA stability of these genes at the posttranscriptional level by interacting with elongins B and C (21).

Germ line or sporadic mutations of the VHL gene are spread all over its three exons. Missense mutations are most common. Non-sense mutations, micro deletions/insertions, splice site mutations and large deletions are also found (22, 23s). VHL gene mutations are also common in sporadic hemangioblastomas (13).

Phenotypes of VHL are based on the absence (type 1) or presence (type 2) of pheochromocytoma. VHL type 2 is subdivided into three categories: type 2A, type 2B and type 2C. Type 2A VHL has pheochromocytoma with CNS HB, but not with RCC. Type 2B exhibits pheochromocytoma, renal cell carcinoma and CNS HBs. A recent notion is that type 2C disease has only pheochromocytoma, with no other disease (24, 25) (Table 1).

**Table 1.** Clinical classifications and manifestations of VHL disease

**Clinical manifestations**			
	CNS HB	Renal Cell carcinoma	Pheochromocytoma	
**VHL type 1**	+	+	-	
**VHL type 2A**	+	-	+	
**VHL type 2B**	+	+	+	
**VHL type 2C**	-	-	+	

## Clinical features

CNS HB is a relatively rare brain tumor, accounting for 2.0% of primary brain tumors (26). VHL patients often have multiple HBs at various sites. Twenty-five to 30% of CNS HBs are associated with VHL, with 70–75% of them being sporadic. In VHL, 50 - 60 % of the HBs are located in the cerebellum, 40–50% in the spinal cord, 10 - 20 % in the brain stem, and 2 - 4% in the pituitary stalk, whereas sporadic HBs occur predominantly in the cerebellum. CNS hemangioblastoma is the earliest or the second earliest manifestation, and the onset age ranges from 7 to 73 years, with the mean being 29 years (26, 27, 28). Signs and symptoms vary based on the anatomical tumor location, associated edema and cyst, and tumor size. Tumors that become symptomatic and require resection usually grow faster than asymptomatic ones (1). Patients with cerebellar HBs can present with symptoms owing to cerebellar impairment and increased intracranial pressure. These include: gait ataxia (64%), dysmetria (64%), headaches (12%), diplopia (8%), vertigo (8%), and emesis (8%). Patients with spinal HBs can present with symptoms associated with radiculopathy and myelopathy: hypesthesia (83%), weakness (65%), gait ataxia (65%), hyper-reflexia (52%), pain (17%), and incontinence (14%). Patients with brain stem HBs can display symptoms mainly due to both lower cranial nerve impairment and high intracranial pressure: hypesthesia (55%), gait ataxia (22%), dysphagia (22%), hyper-reflexia (22%), headaches (11%), and dysmetria (11%) (29). In rare cases, CNS HBs present by intra-parenchymal or subarachnoid hemorrhage (29). Approximately 5% of patients develop polyglobulia, which can be cured by removing the solid tumor mass (28, 30). Most symptoms do not arise from the solid tumor itself but from the associated rapidly growing cyst or syrinx (29). Therefore, symptoms can occasionally develop rapidly; however, usually they develop slowly (29, 31–33). Growth patterns vary and are categorized as saltatory (70–75 % of growing tumors), linear (5–7%) or exponential (20–25%). Many tumors remain same in size for several years (32). VHL patients are found to have a mean of 8.5 tumors/patient (range, 1 to 33 tumors/patients) at initial evaluation. Mean tumor development is 0.4 new tumors/year and is correlated with age, with more frequent development in the younger patients (31). Performance status (PS) of VHL patients with CNS HBs has been assessed according to the Eastern Cooperative Oncology Group performance status (EOCG PS; 4). This study results revealed that most patients have a low ECOG PS score (PS=0, 1). The mean ECOG PS of patients with a single CNS HB was significantly lower than that of patients with multiple CNS HBs (27). Patients bearing HBs often show polycythemia owing to erythropoietin secreted from HB cells. In familial cases, genetic testing can detect VHL gene mutations in peripheral blood or tumor tissue (22). In sporadic HB, such mutations can be detected only in tumor tissues (13).

## Neuroimaging

**Figure 2.**
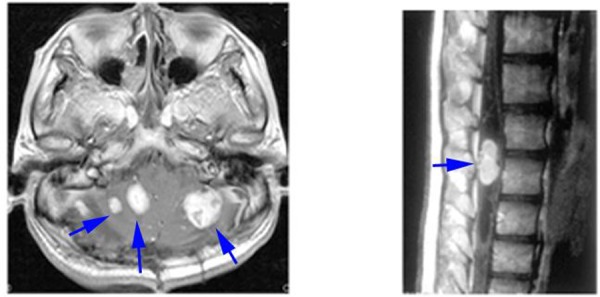
Contrast-enhanced T1 weighted MRI of CNS HBs in VHL. Left, multi-cerebellar HBs; right, lumbar spinal cord HB with syrinx.

HBs are most often visualized by contrast-enhanced T1-weighted MR-imaging (Figure 2). In post-contrast images the tumor tissue appears as a homogenous bright contrast-enhanced mass that clearly stands out from the surrounding tissue. T2-weighted or flair MR-imaging allows excellent quantification of edema and peri-tumoral cysts, which appear as high-signal areas. Cyst walls of HBs are not usually enhanced on MRI. Angiography can be used to highlight the tumor staining, arteriovenous shunting, and early draining veins associated with these tumors prior to resection. Angiography is also performed for intended preoperative embolization in the case of large solid HBs. CT scan has been replaced by MRI (34).

## Clinical diagnostic criteria for VHL

VHL is diagnosed according to clinical diagnostic criteria (29). In the presence of a positive family history, VHL can be diagnosed clinically in a patient with at least one typical VHL tumor, such as retinal or CNS HB, renal cell carcinoma, pheochromocytoma or pancreatic tumor. Endolymphatic sac tumors and multiple pancreatic cysts suggest a positive carrier. In contrast, in patients with a negative family history of VHL-associated tumors, diagnosis of VHL can be made when such patients exhibit two or more CNS HBs or a single HB in association with a visceral tumor such as renal cell carcinoma, pheochromocytoma or pancreatic tumor (29).

## Management and follow-up

### Therapeutic strategy

**Figure 3.**
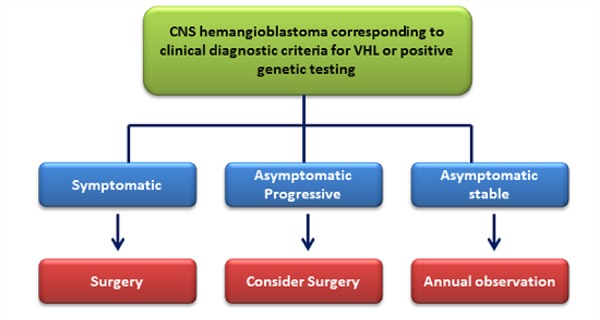
Clinical management of hemangioblastomas.

The therapeutic strategy for each CNS HB in VHL has to be discussed individually with respect to the tumor location, tumor size or associated cysts, as well as symptoms and general condition of the patient, because most VHL patients will develop numerous HBs growing at different rates and at several locations (Figure 3). In addition, a past therapeutic history of each VHL patient should be taken into account. Although the appropriate treatment strategies for CNS HBs are still a matter of debate, there is a general consensus that the symptomatic tumors should be treated (34–38). Since CNS HBs do not grow continuously at the same rate but with intermittent quiescent and rapid-growth phases, therapeutic strategies for asymptomatic tumors in VHL patients are controversial. Asymptomatic tumors, which are stable in MRI screening, are recommended to be followed radiographically. In the case of asymptomatic but progressive tumors, treatment strategies slightly differ in the literature. Some reports recommend early surgery (39) since preoperative neurological symptoms are usually reversible, and surgical resection can be usually performed with low morbidity. For spinal cord HBs, the surgical outcome of the tumor volume less than 500mm^3^ was better than that were larger than 500mm^3^. If the tumor volume exceeds 500mm^3^ during follow-up by MRI, surgical treatment might be considered.

### Preoperative management

As to preoperative management for CNS HBs, preoperative embolization can be helpful in the case of large solid tumors to prevent intraoperative hemorrhage. There is no general consensus on preoperative embolization since this procedure is occasionally associated with side effects such as swelling, hemorrhage, and infarction. The time span between embolization and an operation should not exceed three days, since peri-focal swelling can cause enhanced unnecessary risks (1, 34).

### Surgical treatment

Surgical treatment is usually the first choice therapy for CNS HBs, and its final goal is the complete resection of all tumor components. Since most VHL patients bear multiple CNS HBs and undergo multiple surgeries causing deterioration of performance status (27), at the removal of symptomatic tumors any small asymptomatic tumors in the same anatomical location should be removed simultaneously if they can be found. The cystic wall without contrast enhancement may be left untreated since the cyst wall does not include the tumor cells. The cystic wall usually consists of reactive gliosis without an epithelial lining (38). Occasionally, cysts associated with tumors will refill again in the case of incomplete resection of the solid tumor (40). Since hemangioblastomas are highly vascular tumors, it is not recommended to cut the tumor into pieces since debulking of the tumor can cause extensive bleeding. Without losing sight of the tumor margin, resection must be carried out with careful dissection, and cutting and coagulation of each feeding vessel must be done. It is therefore necessary to consequently dissect the plane between the tumor capsule and the surrounding tissue. In many cases the cyst is much bigger than the solid part and is causative of progressive neurological symptoms (37, 38). The solid tumor itself can be distinguished from the surrounding brain tissue due to its reddish or orange color and can usually be removed completely. However, distinction from the surrounding vessels is occasionally difficult. In this case, intraoperative indocyanine green (ICG) video angiography and fluorescent visualization with 5-ALA facilitate to visualize tumors themselves and/or the surrounding vessels (41, 42). Doppler flow sonography with a contrast-enhancing agent can be also useful, since it is a sensitive intraoperative tool to guide the surgical approach and resection (43, 44).

Motor-evoked potentials for spinal cord HBs should be applied in the case of surgery of spinal cord HBs (38). If the spinal cord HB is not visible on the surface of the spinal cord, enlarged arterialized veins can be helpful for finding the tumor. These enlarged arterialized veins except for those penetrating the tumor should be preserved to avoid swelling and hemorrhage from the tumor. Even if a dorsal fascicle is involved in the tumor, it can usually be removed with no neurological deficit or only slight disturbance of deep sensation (38).

### Radiotherapy

Stereotactic radiosurgery for HBs results in a high local control rate in CNS HBs with acceptable levels of radiation-induced complications (45). Principally, stereotactic radiosurgery can be used for surgically inaccessible or multiple cranial and spinal tumors (46). More recently, fractionated external beam radiotherapy (EBRT; 47) and infratentorial craniospinal radiation therapy (ICSRT; 48) have been investigated for use against CNS HBs, and favorable outcomes were reported.

## Follow-up of CNS HBs in VHL patients

VHL patients with CNS HBs should undergo MRI of the brain and spinal cord at least once a year. VHL patients above 10 years old, who do not display CNS HBs, should undergo MRI screening of their whole neuro-axis every two years. An annual ophthalmoscopy should be performed to screen for retinal HBs. A yearly MRI of the abdomen is recommended to screen for renal cell carcinoma, pancreatic lesions, and pheochromocytoma (29, 34). In addition, a yearly abdominal ultrasound with triennial computed tomography (CT) imaging for renal cell carcinoma, a yearly audiometry for endolymphatic tumor, and pheochromocytoma investigation by urine analysis (metanephrine - VMA) are recommended. Based upon clinical indication these follow-up modalities should be advanced or extended (49).

## Conclusion

The neurologic morbidity and mortality depend on the location and multiplicity of CNS HBs. Because of the complexities of VHL, a deep understanding of pathological and clinical features of HB in VHL is essential, and the management strategies should be tailored to the needs of the individual patients.
